# Transcriptomic Analysis of Flower Blooming in *Jasminum sambac* through *De Novo* RNA Sequencing

**DOI:** 10.3390/molecules200610734

**Published:** 2015-06-10

**Authors:** Yong-Hua Li, Wei Zhang, Yong Li

**Affiliations:** 1 College of Forestry, Henan Agricultural University, Zhengzhou 450002, China; E-Mail: liyhhany@163.com; 2 College of Life Sciences, Xinyang Normal University, Xinyang 464000, China; E-Mail: chawenhua2009@163.com

**Keywords:** flower blooming, transcriptome, RNA sequencing, *Jasminum sambac*

## Abstract

Flower blooming is a critical and complicated plant developmental process in flowering plants. However, insufficient information is available about the complex network that regulates flower blooming in *Jasminum sambac*. In this study, we used the RNA-Seq platform to analyze the molecular regulation of flower blooming in *J. sambac* by comparing the transcript profiles at two flower developmental stages: budding and blooming. A total of 4577 differentially-expressed genes (DEGs) were identified between the two floral stages. The Gene Ontology and the Kyoto Encyclopedia of Genes and Genomes pathway enrichment analyses revealed that the DEGs in the “oxidation-reduction process”, “extracellular region”, “steroid biosynthesis”, “glycosphingolipid biosynthesis”, “plant hormone signal transduction” and “pentose and glucuronate interconversions” might be associated with flower development. A total of 103 and 92 unigenes exhibited sequence similarities to the known flower development and floral scent genes from other plants. Among these unigenes, five flower development and 19 floral scent unigenes exhibited at least four-fold differences in expression between the two stages. Our results provide abundant genetic resources for studying the flower blooming mechanisms and molecular breeding of *J. sambac*.

## 1. Introduction

*Jasminum sambac* (L.) Ait. (Oleaceae) is a small erect or climbing shrub that is native to Bhutan and India. This shrub is cultivated as an ornamental plant worldwide for its attractive and sweetly fragrant flowers. The species grows up to 0.5 to 3 m in height. The leaves are ovate, and the phyllotaxy is opposite or in whorls of three. The flowers bloom from May to August and are produced in clusters of three to 12 together at the ends of branches. To date, no pollination studies have been reported on *J. sambac*. This species has a few morphological and biological characteristics adapted to cross-pollination; these characteristics include large and white petals and strong and sweet fragrances. Previous studies on *J. sambac* have mainly focused on its aromatic compounds [1,2,3], medicinal value [4,5,6] and cultivation physiology [7,8,9]. Molecular biology studies on this species are rare. Two studies cloned three genes involved in the biosynthesis of aromatic volatiles. These genes are deoxyoxylulose-5-phosphate synthase [10], fatty acid hydroperoxide lyase and germacrene D synthase [11].

The transition to blooming is a critical phase switch from vegetative growth to reproductive growth in flowering plants, which ensures sexual reproduction and subsequent generation development. Endogenous and environmental signals initiate a complex network of genetic pathways to activate blooming. The molecular regulatory mechanism underlying flower development has been extensively studied in model plants, such as Arabidopsis and Antirrhinum [12]. A few transcriptomic studies in recent years have investigated the molecular regulation of flower development in non-model plants, such as *Litchi chinensis* [13] and *Nelumbo nucifera* [14]. However, these studies have focused more on the molecular regulation of floral initiation than blooming. Flower blooming is also a key developmental stage, especially in out-crossing flowering plants. Transcriptome sequencing is an efficient method to provide extensive data in a short period with enormous depth and coverage. Such data are important to understand the development processes in plants [13,14,15].

In the present study, the RNA-Seq platform based on the Illumina sequencing system was used to characterize the transcriptomic response during flower blooming by comparing the different transcriptomes at two developmental stages of *J. sambac.* This study aims to characterize the transcriptomes of *J. sambac* and increase the genetic resources available for the genetic or breeding analysis of *J. sambac*.

## 2. Results and Discussion

### 2.1. RNA-Seq and Assembly

**Figure 1:**
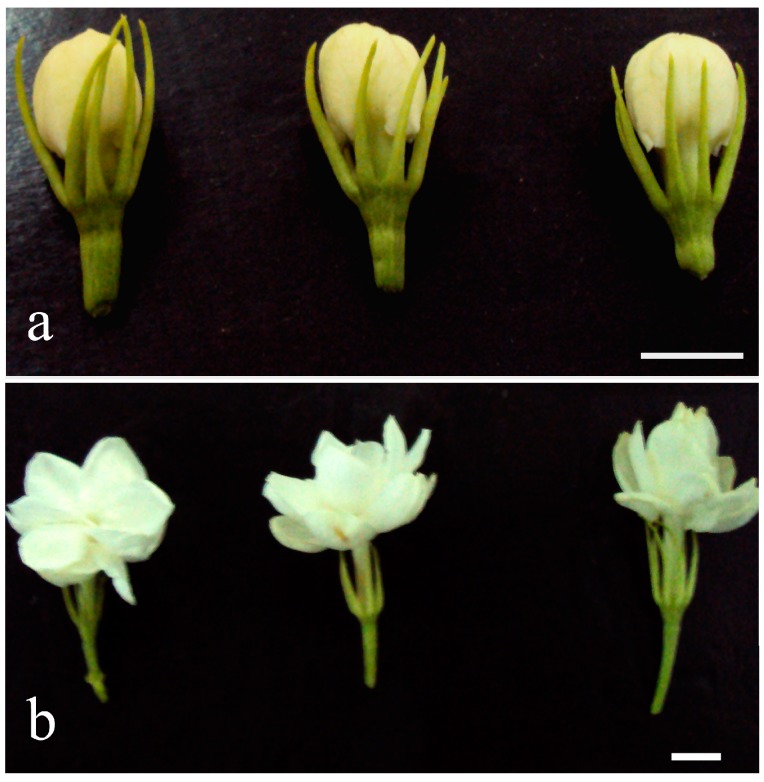
Samples used in this work. (**a**) Samples from three individuals at the flower budding stage (T1); (**b**) Samples from three individuals at the flower blooming stage (T2). Bars: 5 mm.

To understand the molecular basis of flower blooming in *J. sambac*, the flower budding stage (T1) and flower blooming stage (T2) were used to build two libraries for high-throughput sequencing (Figure 1). After cleaning and quality checks, the two libraries produced 3975 and 4570 MB of raw data (Table 1). The phred quality score of >30 (Q30) and guanine-cytosine (GC) percentages of the two libraries were 91.36% and 45.27% and 90.51% and 44.64%, respectively. Approximately 42 million reads (about 19.68 and 22.67 million for T1 and T2, respectively) were obtained from the total RNA-Seq data. Assembly of reads resulted in 113,394 transcripts and 49,772 unigenes with mean sizes of 1425 and 846 bp, respectively (Table 2 and Supplementary Material 1).

**Table 1:** Summary statistics of clean reads in the two libraries.

	Stage T1	Stage T2
Read Number	19,681,589	22,671,385
Base Number	3,975,056,593	4,569,637,577
GC Content	45.27%	44.64%
Q30	91.36%	90.51%

**Table 2:** Summary statistics of the sequence assembly.

Length Range	Contig	Transcript	Unigene
200–300	3,688,028 (98.93%)	19,287 (17.01%)	16,596 (33.34%)
300–500	14,475 (0.39%)	15,583 (13.74%)	11,230 (22.56%)
500–1000	11,261 (0.30%)	19,231 (16.96%)	8681 (17.44%)
1000–2000	9133 (0.24%)	29,990 (26.45%)	7938 (15.95%)
2000+	4944 (0.13%)	29,303 (25.84%)	5327 (10.70%)
Total Number	3727,841	113,394	49,772
Total Length (bp)	184,557,344	161,608,199	42,105,276
Mean Length (bp)	49.51	1425.19	845.96

### 2.2. Transcriptome Functional Annotation

**Figure 2:**
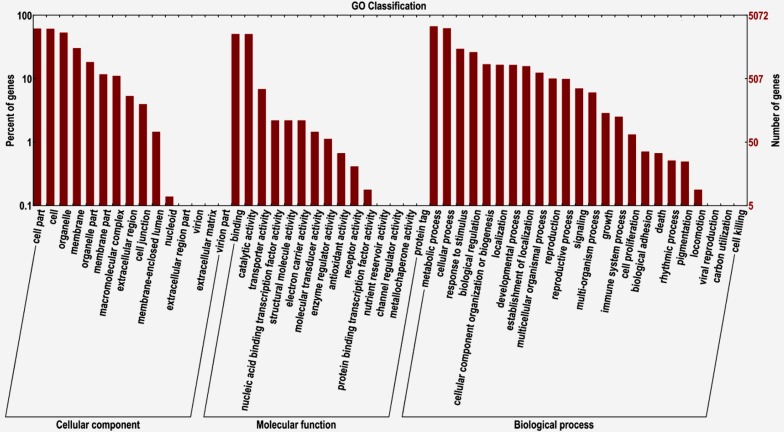
Comparison of GO terms distributions in *J. sambac*.

No closely-related species genome has been reported to date. The genes of this species were annotated as transcriptome without genome. A total of 25,131 unigenes were annotated by searching in the non-redundant (Nr) protein, Swiss-Prot, Kyoto Encyclopedia of Genes and Genomes (KEGG), Cluster of Orthologous Groups (COG) and Gene Ontology (GO) protein databases. The Nr, Swiss-Prot, KEGG, COG and GO protein databases identified 25,110, 327, 5750, 8236 and 5074 unigenes, respectively. GO functional classification was performed using Blast to understand the distribution of the function of unigenes at the macro level. The results showed that these unigenes were assigned to a total of 54 subgroups: 15 subgroups in the “cellular component” group, 15 in “molecular function” and 24 in “biological process” (Figure 2). The top five largest subgroups containing the most unigenes were “metabolic process”, “cell”, “cell part”, “organelle” and “binding” (Figure 2). Pathway analysis with KEGG annotation indicated that these unigenes were involved in 131 pathways (Supplementary Material 2). The highest levels of gene representation were found in “ribosome”, followed by “protein processing in endoplasmic reticulum” and “spliceosome”.

### 2.3. Differentially-Expressed Genes

The false discovery rate (FDR) ≤ 0.01 and the absolute value of log_2_ ratio ≥2 served as the criteria to screen differentially-expressed genes (DEGs). A total of 4577 DEGs (9.20%) were identified between the two floral stages. Among these DEGs, 2292 were upregulated and 2282 were downregulated at T2 compared with T1; 3638 of the 4577 DEGs could be identified in the five protein databases (Supplementary Material 3). The GO annotation of DEGs showed that these genes were assigned to 44 subgroups: 12 in the “cellular component” group, 11 in “molecular function” and 21 in “biological process”. The top five largest subgroups containing the most DEGs were “metabolic process”, “cellular process”, “catalytic activity”, “cell part” and “cell” (Supplementary Material 4). GO enrichment analysis indicated that significant differences in the DEGs between the two stages in “biological process: oxidation-reduction process” (*p* = 0.0008) and “cellular component: extracellular region” (*p* = 0.0287). KEGG annotation of DEGs indicated that these genes were involved in 111 pathways (Supplementary Material 5). The highest levels of gene representation were found in “ribosome”, followed by “plant hormone signal transduction” and “starch and sucrose metabolism”. KEGG enrichment analysis indicated that the DEGs significantly differed in “steroid biosynthesis” (*p* = 0.0045), “glycosphingolipid biosynthesis-globo series” (*p* = 0.0248), “plant hormone signal transduction” (*p* = 0.0380) and “pentose and glucuronate interconversions” (*p* = 0.0407).

### 2.4. Manual Identification of Flower Development and Floral Scent Genes

To identify flower development genes in *J. sambac*, we used the unigene sequences in the BLAST searches of the five protein libraries. A total of 103 unigenes had sequence similarities to the known flower development genes from other plants (Supplementary Material 6). However, most of the flowering development genes (98 of 103) identified in the present study showed no significant differences. The five DEGs were CLAVATA2 (CLV2; c33279.graph_c0), minichromosome maintenance protein 3 (MCM3; c32310.graph_c0), minichromosome maintenance protein 5 (MCM5; c34031.graph_c0), Tesmin/TSO1-like CXC2 (TCX2; c28717.graph_c0) and AGAMOUS-like 15 (AGL15; c31056.graph_c0), which were all downregulated when compared T2 with T1 (Supplementary Material 6). Previous studies reported that the aromatic constituents of *J. sambac* were more than 90 compounds [2]. In this study, we searched the genes that were related to the synthesis of 15 aromatic compounds with the highest relative content in *J. sambac* [2]. A total of 92 floral scent unigenes were found, 19 of which were identified as DEGs (nine upregulated and 10 downregulated; Table 3).

### 2.5. Discussion

Transcriptome analysis is a powerful tool that enables gene discovery and improves our understanding of the molecular regulatory mechanisms of plants under different developmental stages or different growth conditions [16,17,18]. This method has been recently employed in studies on flower development [14,15]. Flower development is an important process throughout the life cycle of seed plants; this process targets successful fertilization and propagation of the subsequent generation. Previous studies on flower development have focused on floral initiation, which determines floral organ or controls blossom time [19]. However, flower blooming is also a highly coordinated event that ensures sexual reproduction, and this stage is accompanied by the enlargement of floral organ, maturity of pistil and stamen, a change in flower color and the emission of floral scent. To date, only one study has conducted the transcriptome analysis of flower blooming in *Rosa chinensis* [20]. The present study used RNA-Seq technology to profile the transcriptome of *J. sambac* and obtained approximately 42 million reads. A total of 25,131 unigenes were successfully annotated against five public protein databases, suggesting their relatively conserved functions. These unigenes were then assigned to 54 GO subgroups and 131 pathways, which indicated the complex regulatory machinery involved in flower blooming. Although only petals of *J. sambac* were selected as the material, our results suggested that a large number of genes were involved in this process. Analyses of GO categories revealed that the five largest subgroups containing the most unigenes were “metabolic process”, “cell”, “cell part”, “organelle” and “binding”. Three of these subgroups were in the “cellular component”, which suggested that the main changes between the two stages resulted from the enlargement of cell size or the increase in cell number. Pathway analysis with KEGG annotation indicated that the highest levels of gene representation were found in “ribosome”, “protein processing in endoplasmic reticulum” and “spliceosome”. Two of them were related to the synthesis and processing of proteins.

To isolate DEGs between T1 and T2, 4577 DEGs were identified. The GO annotation of DEGs showed that the top five largest subgroups were “metabolic process”, “cellular process”, “catalytic activity”, “cell part” and “cell”. Three of these subgroups were related to the cellularity, which further confirmed that the main changes between the two stages resulted from the enlargement in cell size or the increase in cell number. This result is consistent with the GO analyses of all unigenes. KEGG enrichment analysis indicated significant differences in the DEGs between the two stages in “steroid biosynthesis”, “glycosphingolipid biosynthesis-globo series”, “plant hormone signal transduction” and “pentose and glucuronate interconversions”. The pathways associated with flower development, such as “steroid biosynthesis”, “plant hormone signal transduction” and “pentose and glucuronate interconversions”, had also been found in other plants [21,22,23,24]. However, previous studies have not reported the involvement of “glycosphingolipid biosynthesis-globo series” in flower development. The current study is the first to report that pathways show significant differences during flower development. Unigenes related to flower development were manually identified in this study. However, most of the flowering development-related genes were non-DEGs. The five DEGs related to flower development (CLV2, MCM3, MCM5, TCX2 and AGL15) might be involved in flower blooming. The CLV2 gene regulates both meristem and organ development in Arabidopsis [25]. MCM3 and MCM5 are abundantly expressed in flower buds in Arabidopsis, which are related to the DNA replication [26,27]. In Arabidopsis, TCX2 regulates cell proliferation and differentiation during flower development [28]. AGL15 as a member of the MADS regulatory factors can delay senescence and increase floral organ longevity [29]. These findings provide new research directions that might deepen our understanding of blooming regulation in *J. sambac*.

Few aromatic compounds are released at the bud stage in *J. sambac.* However, the release quantity of compounds increases rapidly at the blooming stage [1]. The release mechanism of this floral scent is probably to attract pollinators. In this study, we identified 92 candidate floral scent genes related to 15 aromatic compounds with the highest content. Most of them (73 of 92) did not express significant differences between two stages. The remaining 19 unigenes were DEGs: three related to linalool, one to alpha.-farnesene, one to alpha.-caryophyllene, two to 3-hexen-1-ol, benzoate, three to acetic acid, phenylmenthyl ester, five to methyl salicylate, one to benzyl benzoate, one to indole and two to benzyl alcohol. The 3-hexen-1-ol, benzoate, acetic acid, phenylmenthyl ester, linalool, alpha-farnesene and methyl salicylate had the top five highest content of the aromatic compounds in *J. sambac*. Our results explained the significant aromatic compound differences between the two stages.

**Table 3:** Summary statistics of floral scent genes related to the synthesis of 15 aromatic compounds. The data of 15 aromatic compounds relative content come from Liu *et al.* [2].

Category of Floral Scent	Aromatic Constituents	Relative Content	No. of Genes	Gene Annotation (Gene ID, Red Letters: Upregulated; Green Letters: Downregulated)
Terpenoids	1. linalool	20.1002	4	nerolidol/linalool synthase 2 (c19704.graph_c0);
				nerolidol/linalool synthase 1 (c10900.graph_c0);
				linalool/myrcene synthase (c47600.graph_c0);
				(*E,E*)-geranyllinalool synthase (c22241.graph_c0)
	2. alpha-farnesene	15.3245	43	farnesyltransferase (c29468.graph_c0, c19601.graph_c0, c19799.graph_c0, c18836.graph_c0, c28581.graph_c0, c21749.graph_c1, c44587.graph_c0, c21456.graph_c0, c19099.graph_c0, c26508.graph_c1, c23779.graph_c0, c27539.graph_c0, c19768.graph_c0, c18630.graph_c, c17297.graph_c0, c19238.graph_c0, c13627.graph_c0, c20898.graph_c0, c27921.graph_c0, c26781.graph_c0, c12589.graph_c0, c29535.graph_c0, c8542.graph_c0, c1513.graph_c0, c9983.graph_c0, c28404.graph_c0, c19351.graph_c0, c25991.graph_c0, c27643.graph_c0, c13707.graph_c0, c23982.graph_c0, c29287.graph_c0, c10574.graph_c0, c21749.graph_c0, c40793.graph_c0, c24574.graph_c0);
				farnesyl pyrophosphate synthase (c27053.graph_c0);
				farnesyl-diphosphate farnesyltransferase (c30449.graph_c0, c15924.graph_c0, c36413.graph_c0, c30433.graph_c0, c26200.graph_c0, c30372.graph_c0);
				farnesyl diphosphate synthase (c22132.graph_c0)
	3. Hexanoic acid, 3-hexenyl ester	2.4303	0	-
	4. alpha-caryophyllene	2.3001	1	terpene synthase 3 (c29392.graph_c0)
	5. Germacrene D	1.5928	2	bicyclogermacrene synthase (c35308.graph_c0); germacrene-D synthase (c15826.graph_c1)
Benzenoids/Phenylpropanoids	6. 3-Hexen-1-ol, benzoate	30.4966	8	*o*-succinylbenzoate-CoA ligase (c29960.graph_c0, c11812.graph_c0, c32591.graph_c0, c28597.graph_c0, c35614.graph_c0)
				hexaprenyldihydroxybenzoate methyltransferase (c24032.graph_c0)
				(Z)-3-hexen-1-ol acetyltransferase (c30458.graph_c0, c30940.graph_c0)
	7. Acetic acid, phenylmenthyl ester	29.4231	8	phenylalanine ammonia-lyase (c15788.graph_c0, c30394.graph_c0, c16474.graph_c0, c15481.graph_c0, c11285.graph_c0);
				phenylacetaldehyde reductase (c30130.graph_c0, c29940.graph_c0,
				c5943.graph_c0);
	8. Methyl salicylate	8.2246	8	methyl salicylate esterase (c30688.graph_c0, c3125.graph_c0, c29061.graph_c0, c17812.graph_c0, c31454.graph_c0, c11814.graph_c0, c30703.graph_c0);
				Salicylate *O*-methyltransferase (c32548.graph_c0);
	9. Benzyl benzoate	7.8029	3	phenylcoumaran benzylic ether reductase (c22406.graph_c0, c28180.graph_c0, c9894.graph_c0);
	10. Indole	6.5240	3	Indole-3-acetic acid-amido synthetase (c11944.graph_c0, c25369.graph_c0, c25323.graph_c0)
	11. Benzoic acid, cyclohexyl ester	5.2911	0	-
	12. Salicylic acid, 3-hexenyl ester	2.4519	1	-
	13. Benzoic acid, 2-hydroxy-, phenyl	2.0409	0	-
	14. Benzoic acid, methyl ester	1.7692	0	-
	15. Benzyl alcohol	1.5769	7	benzyl alcohol *O*-benzoyltransferase (c17166.graph_c0, c8299.graph_c0, c26169.graph_c0, c30012.graph_c0); benzyl alcohol benzoyl transferase (c18321.graph_c0, c30940.graph_c0, c24915.graph_c0)

## 3. Experimental Section 

### 3.1. Plant Material

*J. sambac* plants were bred in the trial plot of the Ornamental Horticulture Laboratory at Henan Agricultural University under the same cultivation conditions. Two stages were sampled for transcriptomic sequencing: flower bud stage (T1) and flower blooming stage (T2) (Figure 1). Samples at the T1 stage were collected at 18:00 h, and samples at the T2 stage were collected at 18:00 h the next day. The petals of each stage were sampled from three comparable plants using three biological replications. All samples were immediately frozen in liquid nitrogen and stored at −80 °C for RNA extraction.

### 3.2. RNA Isolation, Library Construction and Sequencing

Total RNA of each sample was isolated using a Quick RNA isolation kit (Bioteke Corporation, Beijing, China) according to the manufacturer’s protocol and treated with RNase-free DNase I (Takara, Dalian, China) to remove genomic DNA contamination. Thereafter, RNA was characterized on a 1% agarose gel and examined with a NanoDrop ND1000 spectrophotometer (NanoDrop Technologies, Wilmington, DE, USA). The RNA integrity number values of these samples were assessed using a 2100 Bioanalyzer RNA Nano chip device (Agilent, Santa Clara, CA, USA). The RNA integrity number was greater than 8.0 for all samples. Thirty micrograms of total RNA were pooled in equal amounts from the three biological replicates for subsequent RNA-Seq.

The construction of the libraries and the RNA-Seq were performed by the Biomarker Biotechnology Corporation (Beijing, China). Poly (A) mRNA was purified from total RNA using oligo (dT)-attached magnetic beads and then broken into short fragments by fragmentation buffer. Taking these cleaved mRNA fragments as templates, the first strand of cDNA was synthesized by priming with random hexamer. The second strand was generated with buffer, dNTPs, RNase H and DNA polymerase I. The double-stranded cDNA fragments were purified with an Agencourt AMPure XP kit (Beckman Coulter, Brea, CA, USA) and resolved with elution buffer (EB) for end repair and the addition of single nucleotide A, and then sequencing adaptors were ligated to the fragments. The suitable size range fragments were selected and purified by AMPure XP beads, and the purified cDNA templates were further enriched using PCR amplification. The cDNA library was sequenced using an Illumina HiSeq 2500 sequencing system (Illumina Inc., San Diego, CA, USA).

### 3.3. Sequence Cleaning, Assembly and Contig Annotation

**Figure 3:**
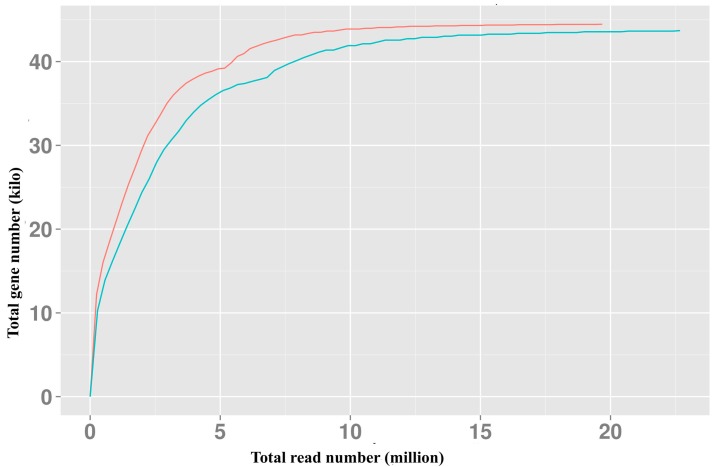
Saturation curve of transcriptomic sequencing reads for *J. sambac*. Red line, samples at the T1 stage; Blue line, samples at the T2 stage.

The raw reads were cleaned by removing adapter sequences, reads with unknown bases greater than 10% and reads with quality scores lower than 20. The left files from all libraries were pooled into one big left.fq file and right files into one big right.fq file. Transcriptome assembly was accomplished based on the left.fq and right.fq using Trinity [30] with min_kmer_cov set to 2 by default and all other parameters set as the default. The contigs with a length less than 200 bp were discarded due to a low annotation rate [31]. The filtered data were deposited in the National Center for Biotechnology Information (NCBI) Sequence Read Archive (SRA) under the Accession Numbers SRX868770 and SRX868796. In this study, about 4 G of sequencing data per sample were finally collected. The present sequencing data were completely saturated [32] and sufficient for subsequent analysis (Figure 3).

Functional annotation was implemented using the program BLAST [33] with an E-value ≤ 10^−5^. The protein databases, including the Nr [34], Swiss-Prot [35], KEGG [36], COG [37] and GO [38] databases, were used for BLAST search and annotation.

### 3.4. Expression Annotation

Gene expression was carried out with RNA-Seq by Expectation-Maximization (RSEM) software [39] bundled with the Trinity package. The gene expression level was calculated using the fragments per kilobase of transcript per million mapped (FPKM) method [40]. The FPKM method eliminates the influence of different gene lengths and sequencing discrepancies on the quantification of gene expression to enable the direct comparison of gene expression between samples. For gene expression difference analysis, Benjamini–Hochberg’s method [41] was used to correct the *p*-values. FDR ≤ 0.01 and the absolute log_2_ ratio ≥ 2 were used as the thresholds to determine the significance of gene expression differences between samples at the T1 and T2 stages. For GO enrichment analysis, topGO [42] was used to identify the statistically overrepresented GO terms for the DEGs. For pathway enrichment analysis, all DEGs were searched for significantly enriched KEGG terms compared with the entire transcriptome background with Fisher’s exact test [43]. 

## 4. Conclusions

This study represents the first broad-scale gene expression study on *J. sambac.* The transcriptome analysis based on the Illumina sequencing system to monitor global transcriptional changes at two flower developmental stages has enabled the comprehensive description of differential transcriptional events during blooming in *J. sambac*. A total of 49,772 unigenes were assembled, and 25,131 unigenes were successfully annotated. These unigenes were assigned to 54 GO subgroups and 131 pathways, which indicated the complex regulatory machinery involved in flower blooming. We also identified 103 flower development candidate genes and 92 floral scent candidate genes. Among these genes, five flower development genes and 19 floral scent genes were DEGs. Notably, transcriptome analysis with more samples at different flower developmental stages and gene function validation using real-time qRT-PCR were necessary for future studies on *J. sambac.*

## Supplementary Materials

Supplementary materials can be accessed at: http://www.mdpi.com/1420-3049/20/06/10734/s1.
